# Angiotensin II Contributes to Renal Fibrosis Independently of Notch Pathway Activation

**DOI:** 10.1371/journal.pone.0040490

**Published:** 2012-07-09

**Authors:** Carolina Lavoz, Raquel Rodrigues-Diez, Alberto Benito-Martin, Sandra Rayego-Mateos, Raúl R. Rodrigues-Diez, Matilde Alique, Alberto Ortiz, Sergio Mezzano, Jesús Egido, Marta Ruiz-Ortega

**Affiliations:** 1 Cellular Biology in Renal Diseases Laboratory. Universidad Autónoma, Madrid, Spain; 2 Dialysis Unit, IIS-Fundación Jiménez Díaz-Universidad Autónoma, Madrid, Spain; 3 Division of Nephrology, School of Medicine, Universidad Austral, Valdivia, Chile; 4 Nephrology and Hypertension, IIS-Fundación Jiménez Díaz-Universidad Autónoma Madrid, Spain; Institut National de la Santé et de la Recherche Médicale, France

## Abstract

Recent studies have described that the Notch signaling pathway is activated in a wide range of renal diseases. Angiotensin II (AngII) plays a key role in the progression of kidney diseases. AngII contributes to renal fibrosis by upregulation of profibrotic factors, induction of epithelial mesenchymal transition and accumulation of extracellular matrix proteins. In cultured human tubular epithelial cells the Notch activation by transforming growth factor-β1 (TGF-β1) has been involved in epithelial mesenchymal transition. AngII mimics many profibrotic actions of TGF-β1. For these reasons, our aim was to investigate whether AngII could regulate the Notch/Jagged system in the kidney, and its potential role in AngII-induced responses. In cultured human tubular epithelial cells, TGF-β1, but not AngII, increased the Notch pathway-related gene expression, Jagged-1 synthesis, and caused nuclear translocation of the activated Notch. In podocytes and renal fibroblasts, AngII did not modulate the Notch pathway. In tubular epithelial cells, pharmacological Notch inhibition did not modify AngII-induced changes in epithelial mesenchymal markers, profibrotic factors and extracellular matrix proteins. Systemic infusion of AngII into rats for 2 weeks caused tubulointerstitial fibrosis, but did not upregulate renal expression of activated Notch-1 or Jagged-1, as observed in spontaneously hypertensive rats. Moreover, the Notch/Jagged system was not modulated by AngII type I receptor blockade in the model of unilateral ureteral obstruction in mice. These data clearly indicate that AngII does not regulate the Notch/Jagged signaling system in the kidney, *in vivo* and *in vitro*. Our findings showing that the Notch pathway is not involved in AngII-induced fibrosis could provide important information to understand the complex role of Notch system in the regulation of renal regeneration vs damage progression.

## Introduction

The Notch pathway is an evolutionarily conserved mechanism involved in the formation of complex structures, such as the kidney [1]–[3], that influences differentiation, proliferation, and apoptotic events at all stages of the development [4]–[6]. The membrane-bound ligands Delta-like-1/3/4 or Jagged-1/2 can bind to the single-pass transmembrane Notch receptors (Notch1/2/3/4) [7], [8]. On activation, the Notch intracellular domain (NICD) is cleaved by γ-secretases and translocates into the nucleus to interact with the transcriptional regulator recombination signal-binding protein-1 for JKappa (RBP-Jκ) and then activate downstream transcription factors, such as hairy-and-enhancer of split 1 (HES-1) and HES-related repressor proteins (Hey), which may mediate the effects on the cell fate [9], [10].

The Notch pathway participates in physiological and pathological processes, including cancer [11], and regeneration of the vasculature [12], [13]. Regarding the kidney, Notch expression is virtually absent in the glomeruli of healthy adult kidneys, while Notch activation is observed in renal progenitors and podocytes of patients with glomerular disorders [14]. Notch ligands and its receptors are expressed in a wide range of renal diseases. Podocyte-specific Notch expression is correlated with albuminuria and glomeruloesclerosis, while expression of cleaved Notch-1 in tubules is associated with tubulointerstitial fibrosis [15]. In transgenic mice, specific Notch activation in podocytes causes chronic glomerular injury and albuminuria [16], [17]. In experimental models of tubulointerstitial damage, activation of Notch is found in tubular epithelial and interstitial cells [5], [18], [19]. However, the beneficial effect of Notch modulation in renal disease progression is still controversial [14], [20], [21].

TGF-β1 is a key profibrotic cytokine that contributes to tubulointerstitial damage and renal fibrosis [22]–[24]. In tubular epithelial cells, TGF-β1 activates the Notch signaling system and stimulates the expression of the Notch ligands Jagged-1, Jagged-2, as well as the receptors Notch-1 and Notch-4, while Notch-2 expression is not affected [18], [25]. In these cells TGF-β1 induces epithelial-mesenchymal transition (EMT) [26]. TGF-β1-induced Jagged-1 overexpression occurs earlier than changes in EMT-associated genes, and blockade of Notch activation markedly diminishes TGF-β1-mediated EMT [27]. Based on these observations, several authors have suggested that the Notch pathway could regulate renal EMT and fibrosis.

Angiotensin II (AngII) plays a key role in the progression of chronic kidney damage, contributing to renal fibrosis. Many *in vitro* and experimental studies have demonstrated that AngII activates renal cells to produce profibrotic factors and extracellular matrix proteins (ECM) [28], [29]. The interrelation between AngII and TGF-β1 is well established. AngII and TGF-β1 share many, profibrotic mediators and intracellular signaling systems [30], [31]. In particular, in tubular epithelial cells both AngII and TGF-β1 can induce EMT [23], [24], [32], and TGF-β1 is known to activate the Notch pathway [18], [25]. For these reasons, our aim was to evaluate the contribution of the Notch/Jagged system to AngII-induced renal responses in this paper. We have identified a signaling mechanism, the Notch pathway, not shared by AngII and TGF-β1, and not involved in AngII-induced fibrosis. Our results may have therapeutic relevance for understanding the complex relation between renal disease progression and regeneration.

## Results

### AngII did not increase Jagged-1 expression in cultured tubular epithelial cells

**Figure 1:**
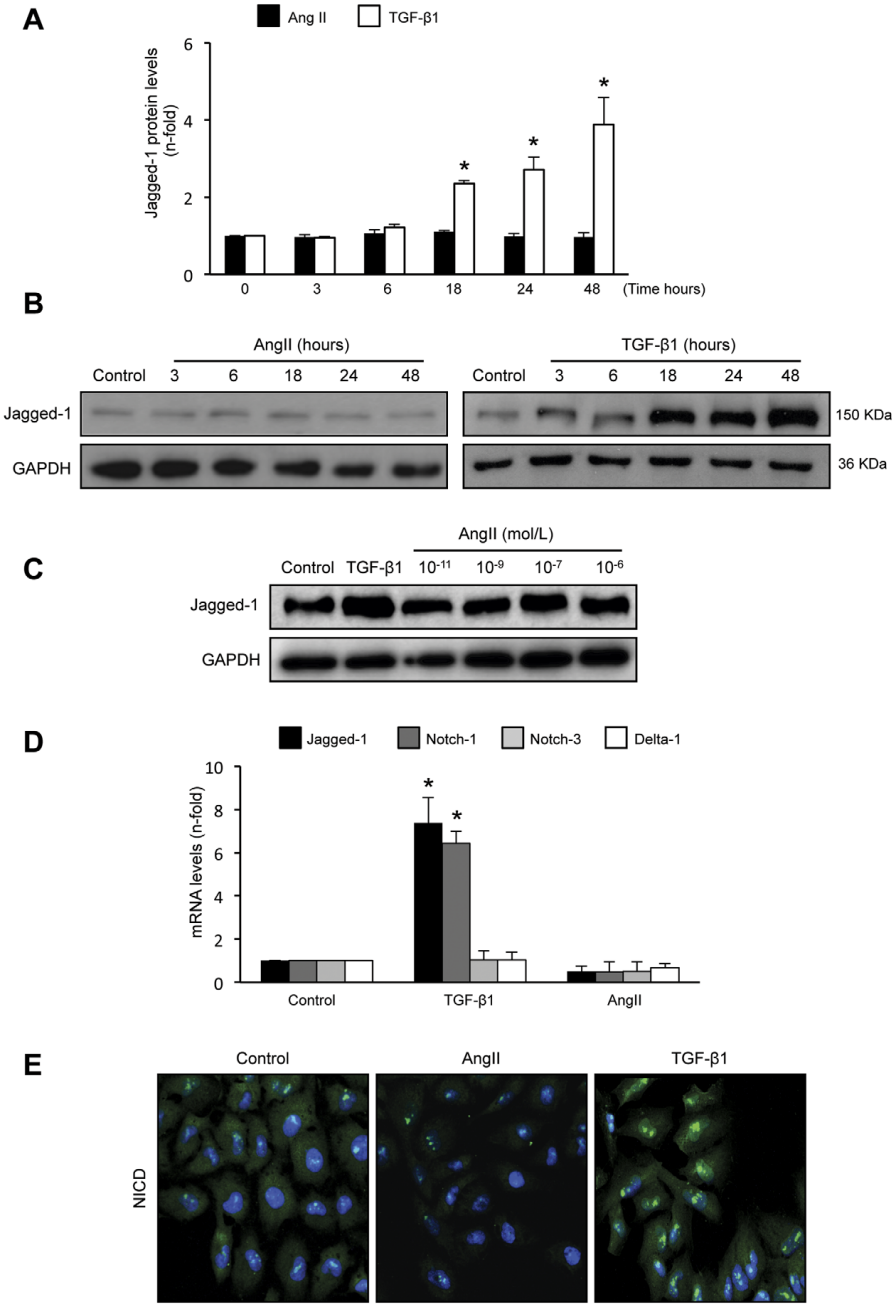
TGF-β1, but not AngII, increased Jagged-1 synthesis in cultured human tubular epithelial cells. Cultured human tubular epithelial cells (HK-2) were treated with 10^−7^ mol/L AngII or 10 ng/mL TGF-β1 for increasing times. **A.** Results of total protein expression were obtained from densitometric analysis and expressed as ratio protein/GAPDH as n-fold over control as mean ± SEM of 3 independent experiments. *p<0.05 vs control. Figure **B** shows a representative Western blot experiment. **C.** **Dose-response of AngII.** HK-2 cells were stimulated with AngII (10^−6^ to 10^−11^ mol/L) for 48 hours and Jagged-1 protein levels were determined by Western blot. Figure shows a representative experiment of 3 done. **D. TGF-β1, but not AngII, upregulated Notch-related genes in tubular epithelial cells.** Gene expression levels of jagged-1, delta-1 and notch1/3 were determined by Real Time PCR. Data are expressed as mean ± SEM of 5 experiments. *p<0.05 vs control. **E.** Nuclear localization of activated Notch-1 (NICD) is only observed in TGF-β1 treated cells for 48 hours (green staining), while in control and AngII-treated cells there is no positive NICD staining. Nuclei are in blue (DAPI staining). Figure shows a representative experiment of 2 done by confocal microscopy. Magnification 200x.

In cultured human tubular epithelial HK-2 cells previous studies have shown that TGF-β1, at doses between 5 and 50 ng/mL, actives Notch pathway and induces EMT changes [27]. Stimulation of HK-2 cells with 10−7 mol/L AngII did not modify protein levels of the Notch ligand Jagged-1, at any time point studied, while TGF-β1 significantly increased Jagged-1 synthesis, starting at 18 hours and remaining elevated up to 48 hours (ure 1A and B). Moreover, incubation with AngII (dose range 10−6 mol/L to 10−11 mol/L) showed no changes in Jagged-1 protein levels (figure 1C). Gene expression analysis of the Notch components showed that only stimulation with TGF-β1, but not AngII, for 24 hours increased Jagged-1 and its receptor Notch-1. In contrast, neither TGF-β1 nor AngII modified Delta-1 and Notch-3 gene levels (figure 1D). By confocal microscopy, activated Notch intracellular domain (NICD) was only detected in the nuclei of TGF-β1-treated cells, while in control or AngII-treated cells there was no NICD immunostaining (figure 1E). These data clearly demonstrated that in tubular epithelial cells TGF-β1, but not AngII, increased the Notch pathway-related gene expression, and activated Notch, determined by Jagged-1 production and NICD nuclear translocation, where it may activate gene transcription, as described [6], [33].

### Activation of the Notch pathway was not involved in AngII-induced EMT in cultured tubular epithelial cells

**Figure 2:**
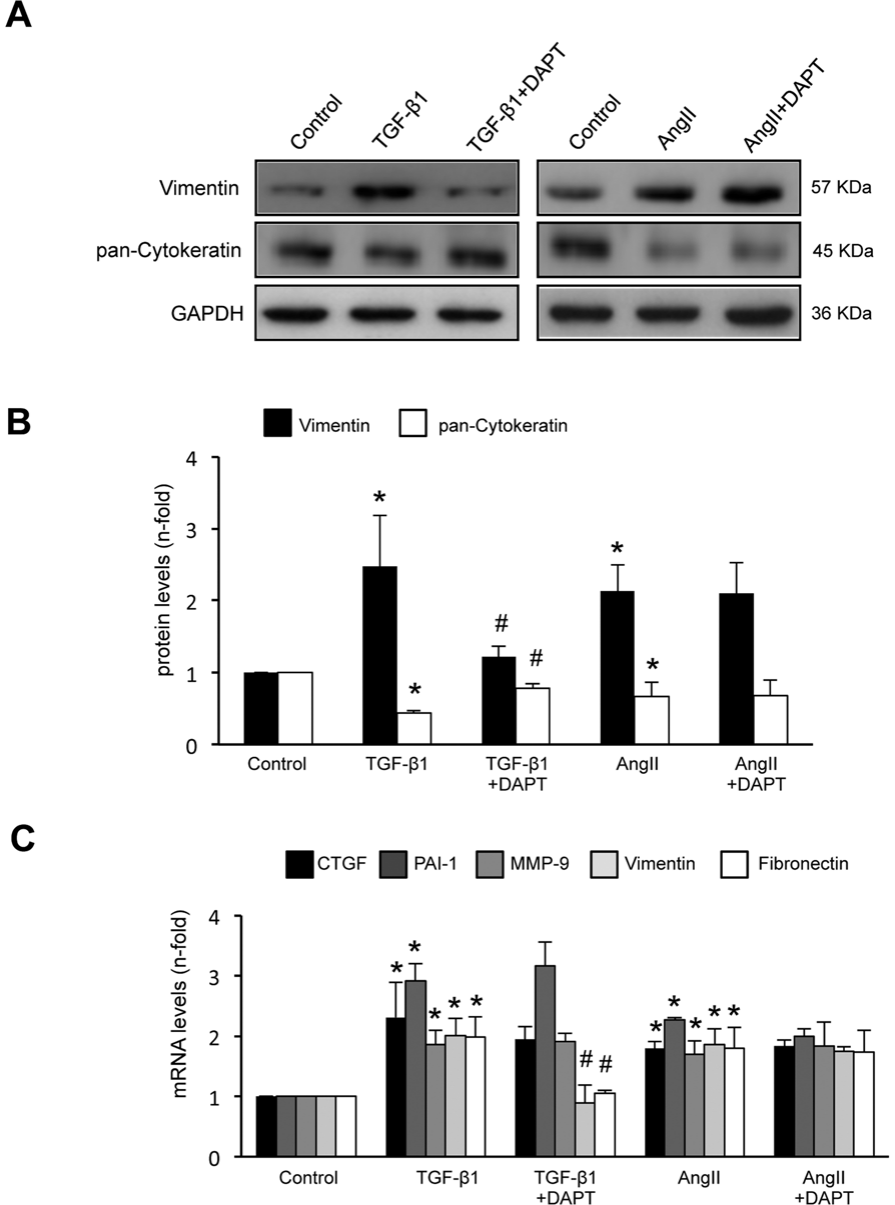
Blockade of the Notch pathway inhibited TGF-β1-, but not AngII- induced EMT changes in cultured tubular epithelial cells. HK-2 cells were pretreated with the gamma-secretase inhibitor, DAPT (3×10^−8^ mol/L) for 1 hour and then stimulated with 10^−7^ mol/L AngII or 10 ng/mL of TGF-β1 for 24 or 48 hours (gene and protein studies, respectively). Figure **A** shows a representative Western blot experiment **B.** Results of total protein expression were expressed as mean ± SEM of 3 independent experiments. Figure **C** shows gene expression levels, determined by Real Time PCR, were shown as mean ± SEM of 5 experiments. *p<0.05 vs control, #p*<*0.05 vs TGF-β1.

In tubular epithelial cells AngII, at the dose of 10^−7^ mol/L, induces EMT changes and upregulation of profibrotic factors and ECM [23], [24], [32], [34], [35]. To evaluate the role of the Notch pathway in AngII-induced profibrotic responses cells were treated with the γ-secretase inhibitor, DAPT, which inhibits the signaling from all Notch receptor types [6]. Preincubation of HK-2 cells for 1 hour with DAPT inhibited TGF-β1-induced EMT phenotypic changes, including induction of the mesenchymal marker Vimentin and downregulation of the adhesion-related molecule Cytokeratin (figure 2), as described [27]. By contrast, DAPT had no effect on any AngII-induced EMT changes (figure 2). Interestingly, Notch blockade did not modify TGF-β1 or AngII-induced changes in other profibrotic factors, including connective tissue growth factor (CTGF), Matrix metallopeptidase-9 (MMP-9) and Plasminogen activator inhibitor-1 (PAI-1) mRNA upregulation (figure 2D).

**Figure 3:**
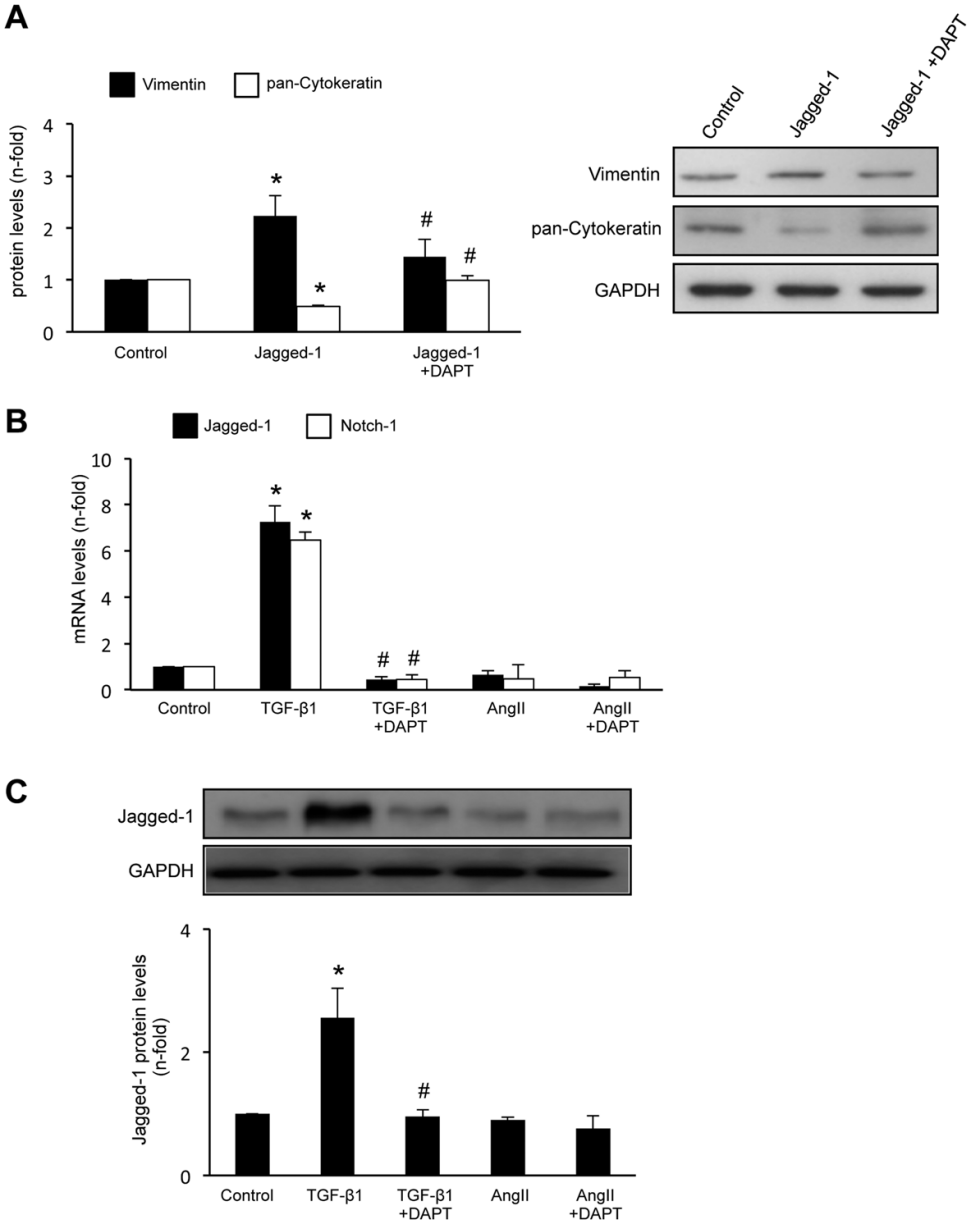
Jagged-1 induced EMT changes in cultured tubular epithelial cells . **A.** HK-2 cells were treated with 50 ng/mL Jagged-1 for 48 hours. Left panel: total protein levels as mean ± SEM of 3 independent experiments. *p<0.05 vs control; #p*<*0.05 vs Jagged-1. Right panel: representative Western blot experiment. **Blockade of the Notch pathway inhibited TGF-β1-induced upregulation of Notch components.** Cells were pretreated for 1 hour with 3×10^−8^ mol/L DAPT and then incubated with 10 ng/mL TGF-β1 or 10^−7^ mol/L AngII for 24 or 48 hours (gene and protein studies, respectively). **B.** Gene expression levels are expressed as mean ± SEM of 5 experiments. Figure **C** shows a representative western blot of Jagged-1 and data as of mean ± SEM of 3 independent experiments. *p<0.05 vs control; #p*<*0.05 vs TGF- β1.

We have further explored the direct role of Jagged-1 in EMT. Incubation of HK-2 cells with Jagged-1 recombinant protein for 48 hours induced phenotypic conversion from epithelial to fibroblast-like morphology (data not shown) and changes in EMT markers (figure 3A). DAPT also diminished TGF-β1-induced upregulation of Notch genes (figure 3B) and Jagged-1 production (figure 3C). All these data supporting the hypothesis that TGF-β1 via Notch pathway activation could regulate EMT in tubular epithelial cells.

### AngII did not increase Jagged-1 expression in cultured podocytes and renal fibroblasts

**Figure 4:**
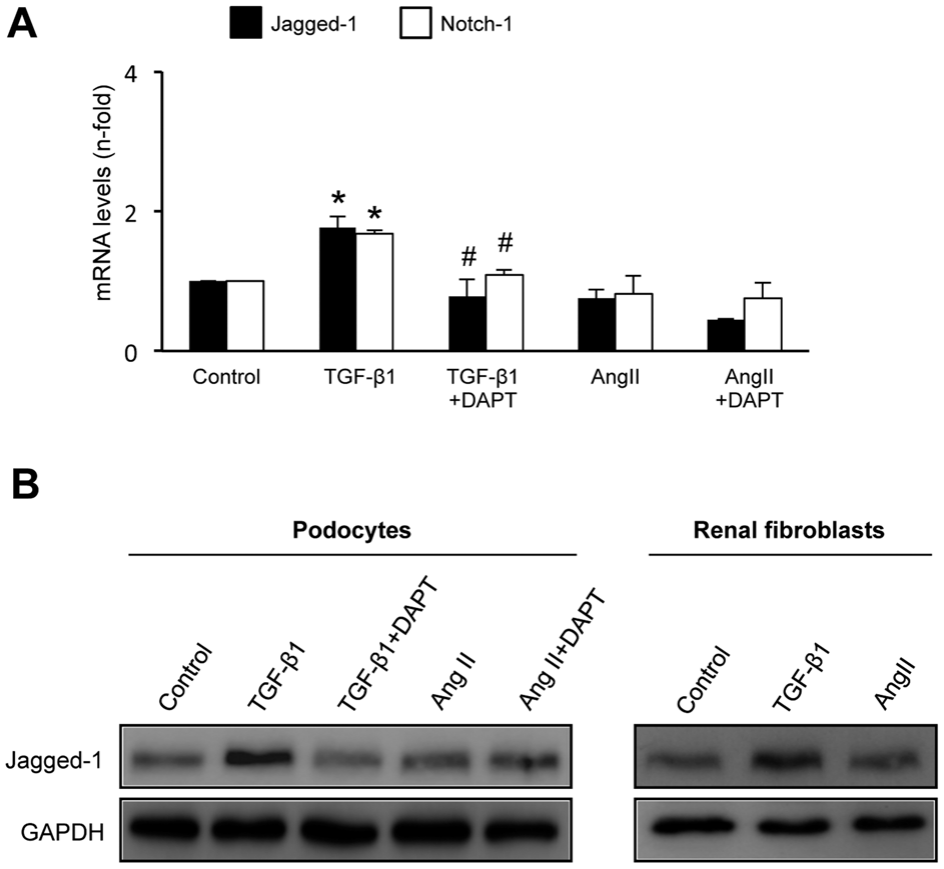
TGF-β1, but not AngII, increased Jagged-1 expression in human podocytes and murine renal fibroblasts. Cells were treated with 10^−7^ mol/L AngII or 10 ng/mL TGF-β1 for 24 or 48 hours (gene and protein studies, respectively). In some points cells were pretreated with the gamma-secretase inhibitor, 3×10^−8^ mol/L DAPT, for 1 hour. **A.** In human podocytes, gene expression levels of Notch components are expressed as mean ± SEM of 5 experiments. *p<0.05 vs control, #p*<*0.05 vs TGF- β1. **B.** Representative Western blot of Jagged-1 levels in podocytes and fibroblasts of 3 independent experiments.

In human podocytes, TGF-β1, but not AngII, upregulated Jagged-1 mRNA (figure 4A) and protein levels (figure 4B). Blockade of Notch activation by DAPT significantly diminished Jagged-1 upregulation by TGF-β1. In murine renal fibroblasts, incubation with TGF-β1, but not AngII, increased Jagged-1 synthesis at 48 hours (figure 4B).

### AngII increased TGF-β1 production in renal cells

**Figure 5:**
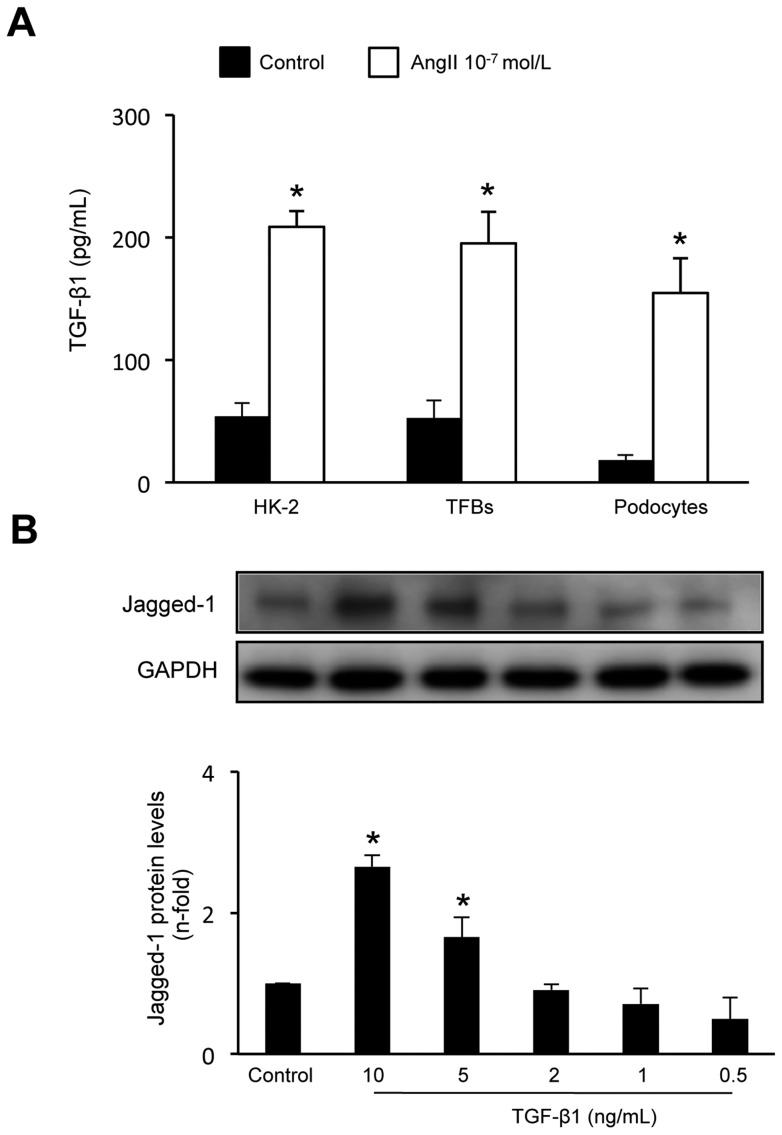
AngII increased TGF-β1 production in renal cells. The different cell types, human tubular epithelial cells (HK-2), murine renal fibroblasts (TFBs) and human podocytes, were treated with 10^−7^ mol/L AngII for 48 hours. Then, supernatants were collected, and active TGF-β1 was determined by ELISA. Figure **A** shows TGF-β1 protein levels as mean ± SEM of 3 independent experiments analyzed by duplicate. *p<0.05 vs control. **B. Low doses of TGF-β1 did not increase Jagged-1 protein production in tubular epithelial cells.** HK-2 cells were stimulated with TGF-β1 (10 to 0.5 ng/mL) for 48 hours and Jagged-1 protein levels were determined by Western blot. Figure shows a representative experiment and data as mean ± SEM of 3 experiments. *p<0.05 vs control.

Previous studies have demonstrated that AngII increased TGF-β1 gene expression and production of active protein [28]–[32]. Next, the potential role of endogenous TGF-β1 production in the activation of Notch pathway was evaluated. After 48 hours of incubation with 10^−7^ mol/L AngII a significant increase in TGF-β1 production was found in the conditioned media of the different cell types evaluated in the present study (HK-2, TFBs and podocytes) (Figure 5A), in the same experiments that AngII did not active the Notch pathway. The amount of active TGF-β1 detected in supernatants was around 200 pg/mL. Therefore, we evaluated whether low doses of TGF-β1 could activate the Notch pathway in renal cells. In HK-2 cells, stimulation with TGF-β1 at low doses (2 ng/mL to 0.5 ng/mL) did not increase Jagged-1 production, while only doses higher than 5 ng/mL activated this pathway (figure 5B), as previously described [27]. These data suggest that although AngII increased active TGF-β1 protein levels, this endogenous TGF-β1 production is not enough to activate the Notch pathway in renal cells.

### The Notch/Jagged signaling system was not activated in the kidney of AngII-infused rats or hypertensive rats

**Figure 6:**
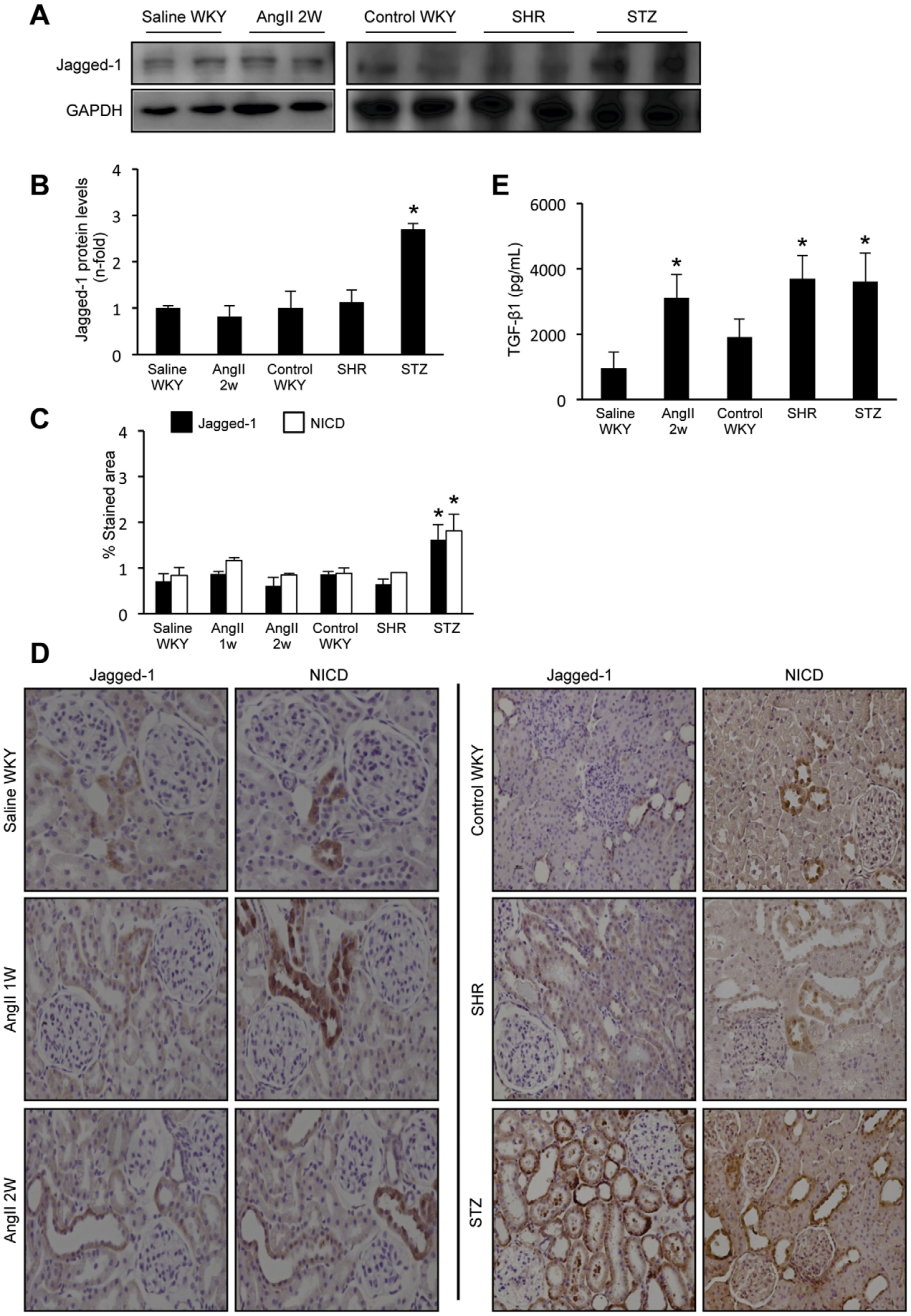
Renal Notch pathway is not upregulated in AngII-infused rats or in SHR. Infusion of AngII (100 ng/kg/min) was done in normotensive rats from 24 hours to 2 weeks, saline infusion was used as control. Spontaneously hypertensive rats (SHR) of 16 weeks were studied, WKY of the same age were used as control, and streptozotocin-induced diabetic rats (STZ), a known model of activated renal Notch. **Renal jagged-1 protein levels were elevated only in STZ rats, but not in AngII-infused or SHR rats.** In total renal extracts, Jagged-1 levels were determined by western blot. Figure **A** shows a representative experiment of 2 animals per group and in **B** data as mean ± SEM of 8 −10 rats per group. *p<0.05 vs control WKY. **Jagged-1 and Notch intracellular domain (NICD) expression were evaluated by immunohistochemistry.** **C**. Quantification of stained area as mean ± SEM of 8–10 animals per group. *p<0.05 vs control WKY. Figure **D** shows a representative picture of each group. Original magnification 200x. **Renal TGF-β1 protein levels were determined by ELISA.** Figure **E** shows data of active TGF-β1 in total renal extracts expressed as mean ± SEM of 4–6 rats per group. *p<0.05 vs its corresponding control group.

To investigate the *in vivo* effect of AngII in the Notch pathway activation in the kidney, the model of systemic infusion of AngII into rats was used. Renal levels of Jagged-1 were not upregulated in response to AngII infusion for 2 weeks compare to saline-infused ones, used as controls (figure 6A and B). By immunohistochemistry, we have confirmed that renal Jagged-1 expression was not changed in response to AngII, at any time point evaluated, up to 2 weeks (figure 6C and D). Notch activation can also be detected by evaluation of NICD levels. In AngII or saline-infused groups NICD immunostaining was similar (figure 6C and D). The onset of renal fibrosis in this model is well characterized. Upregulation of profibrotic genes, including TGF-β1, CTGF and PAI-1, were observed at 3 days. Renal protein levels of CTGF were increased at 3 days, Fibronectin deposition was found 1 week later [36], while TGF-β1 protein levels were not elevated until 2 weeks [32], as we have confirmed here (figure 6E). At 2 weeks sustained overexpression of profibrotic factors and tubulointerstitial fibrosis were observed (data not shown), as described [32], [34].

We further evaluated the role of Notch pathway in hypertension-induced renal damage using the model of spontaneously hypertensive rats (SHR). At 16 weeks of age, SHR rats presented elevated blood pressure, increased proteinuria and urinary albumin (Table 1), TGF-β1 overproduction (figure 6E) and elevated collagen deposition (data not shown), compare to control WKY of the same age. In SHR, renal Notch pathway was not activated, shown similar levels that normotensive WKY rats (figure 6), as described in hypertensive patients with renal injury [15].

**Table 1:** Data of the experimental models of spontaneously hypertensive rats (SHR) and diabetic nephropathy induced by streptozotocin injection (STZ).

	*Systolic BP* (mmHg)	*Protenuria* (mg/24h)	*Urinary Albumin* (µg/24h)
**Control**	112.0±3.9	4.5±1.8	0.44±0.3
**SHR**	143.1±10.2	8.8±3.6	2.00±0.7
**STZ**	121.2±4.8	8.6±3.6	2.31±0.4

An additional control of the experiment was to evaluate whether renal Notch is activated in a rat model of diabetic nephropathy induced by streptozotocin injection (STZ). Previous studies have demonstrated activation of renal Notch in human and experimental diabetic nephropathy [16], [37]. At 6 weeks after induction of diabetic nephropathy, rats presented increased proteinuira and albuminuria (Table 1), elevated renal TGF-β1 protein levels (figure 6E) and fibrosis (not shown). In the immunohistochemistry experiments done in parallel with the other models, the renal samples of the diabetic rats showed a clear up-regulation of Jagged-1 and NICD levels, mainly in tubulointerstitial and glomerular cells (figure 6).

### Blockade of AngII receptors did not regulate Notch/Jagged signaling system in the model of unilateral ureteral obstruction in mice

**Figure 7:**
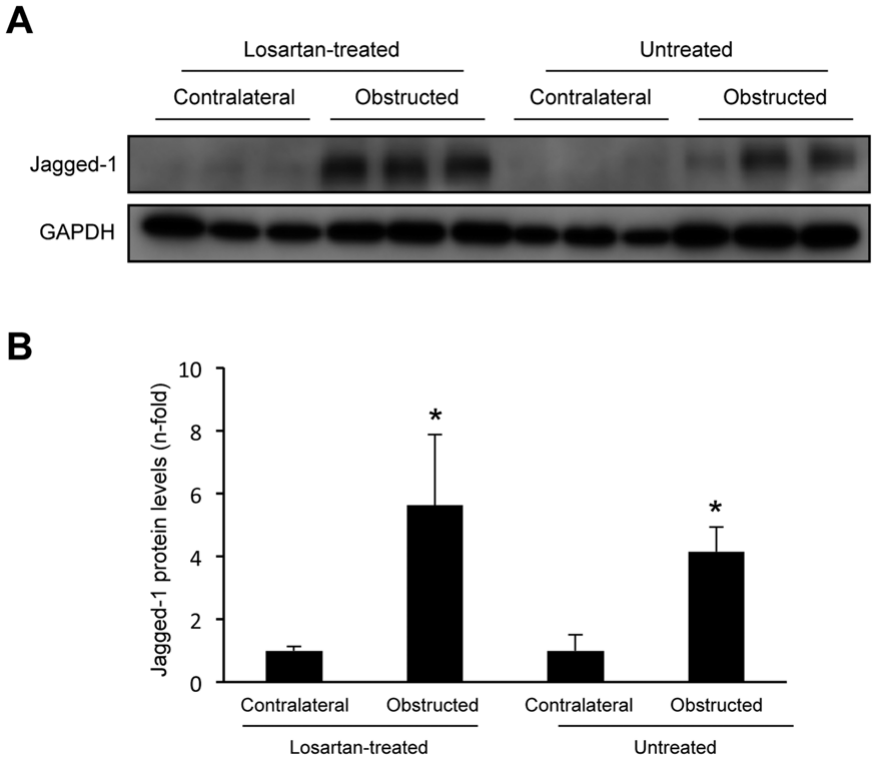
AT1 antagonism increased renal Jagged-1 protein levels in the model of unilateral ureteral obstructed kidneys in mice. The figure **A** shows a representative experiment of Jagged-1 protein levels evaluated by western blot and in **B** data as mean ± SEM of 6 animals per group. *p<0.05 vs contralateral kidneys.

Several groups have shown activation of Notch/Jagged in experimental models of renal damage. Interestingly, microarray analysis discloses that Jagged-1 is one of the most highly expressed genes in the experimental model of unilateral ureteral obstruction (UUO) [5], [18], [19]. Previous studies have demonstrated that AngII plays a key role in the pathogenesis of UUO, and pharmacological blockade of AngII (by ACE inhibitors or AT1 receptor antagonists) ameliorates disease progression [38], [39]. However, there are no studies evaluating whether AngII blockade modulates the Notch pathway in experimental renal diseases. Thus, in UUO model, we have observed that treatment with an AT1 antagonist (losartan, 10 mg/kg/day), ameliorated disease progression, including inhibition of inflammatory cell infiltration and downregulation of MCP-1 gene overexpression (figure S1), and diminution of renal fibrosis (not shown) and TGF-β1 overproduction (figure S1), as previously described [38], [39]. Jagged-1 protein levels were markedly increased in obstructed kidneys compare to contralateral ones, as described [5], [18], [19]. In losartan-treated mice, obstructed Jagged-1 protein levels were similar than in untreated obstructed ones (figure 7). This data further supports the notion that AngII regulates renal fibrosis independently of Notch pathway.

## Discussion

The main finding of this paper is that AngII does not modulate the Notch pathway in the kidney. In *in vivo* studies, we have found that systemic infusion of AngII into rats for 2 weeks, at a dose that caused tubulointerstitial damage and fibrosis, did not upregulate renal expression of activated Notch or Jagged-1, suggesting that the Notch/Jagged pathway is not involved in AngII-induced renal damage. In spontaneously hypertensive rats, studied at the time that presented albuminuria and interstitial fibrosis, renal expression of activated Notch or Jagged-1 was similar to normotensive healthy WKY rats of the same age. In a wide range of kidney diseases, renal activation of the Notch components has been described. However, these authors have observed that in patients with hypertensive nephrosclerosis renal the Notch/Jagged-1 system was not upregulated [15]. Blockade of AngII, by ACE inhibitors or receptor blockers, is one the current clinical therapies that have proven to ameliorate renal disease progression [31]. In the experimental model of UUO, we have found that AT1 antagonist treatment ameliorated renal inflammation and fibrosis, by local inhibition of chemokines and profibrotic factors, including TGF-β1, as described [38], but did not diminish renal Jagged-1 expression. Our *in vitro* studies clearly demonstrated that although TGF-β1 activated the Notch pathway in renal cells [5], [18], [27], AngII did not regulate this system. In cultured human tubular epithelial cells, we have found that AngII did not up-regulate Notch related genes, or increased Jagged-1 protein levels. Similar results were observed in podocytes and renal fibroblasts. These data clearly indicates that the Notch/Jagged signaling system is not involved in renal damage associated to AngII and hypertension.

Many works have shown that the Notch/Jagged signaling is essential for epithelial function and appears to contribute to EMT in embryogenesis and cancer [40], [41]. In cultured tubular epithelial cells, suppression of the Notch pathway by pharmacological inhibition of γ-secretase markedly inhibited phenotypic EMT changes induced by TGF-β1 [25], [27]. In these cells TGF-β1 and AngII induce EMT by common mechanisms, including the Smad pathway and MAPK cascade [32], [34]. Data presented here demonstrated that γ-secretase inhibition did not modulate AngII-induced EMT, illustrating a different mechanism of action between AngII and TGF-β1. Interestingly, in these cells the Notch-1 ligand Jagged-1 induced a transition to a fibroblast-like phenotype and changes in EMT markers, such as loss of epithelial proteins and induction of mesenchymal markers, supporting the importance of Notch/Jagged-1 activation in EMT regulation. However, the contribution of EMT to renal fibrogenesis is a matter of intense debate [24], [41]–[44]. In this sense, in a transgenic mice model, the specific Notch activation in tubular and interstitial cells induced renal damage, characterized by increased cell proliferation of both cell types and fibrosis, but changes in EMT markers were not detected [20].

The relation between AngII and TGF-β1 in fibrosis is well known [28]–[32]. Many studies have demonstrated that TGF-β1 acts as a downstream mediator of AngII-induced renal fibrosis and both factors share several intracellular mechanisms involved in the regulation of ECM accumulation [28], [29]. In tubular epithelial cells, we have demonstrated that although AngII increased active TGF-β1 levels, this endogenous TGF-β1 production is not enough to activate the Notch pathway. This observation support our *in vivo* findings in the models of AngII and hypertension-induced renal damage, both characterized by TGF-β1 overexpression and fibrosis in the absence of Notch pathway activation, as well as by the data in the model of UUO showing the lack of effect on renal Jagged-1 levels, but downregulation of TGF-β1 and renal damage, in response to AT1 antagonism. Interestingly, in a previous study the Notch blockade did not inhibit TGF-β1-induced upregulation of some profibrotic genes, such as CTGF, thrombospondin, MMP-9 [27] and, as described here, PAI-1. We have found that these genes are also upregulated by AngII independently of Notch activation. Importantly, CTGF has been described as a key downstream profibrotic mediator of AngII and TGF-β1 in several cells types, including renal cells [28]. PAI-1 has been involved in AngII-induced vascular fibrosis, independently of TGF-β [45], [46]. These data indicated that several profibrotic-related events induced by TGF-β1 and AngII are independent of the Notch pathway activation.

Podocyte-specific Notch activation severely injures the glomerular filtration barrier in the kidney. Inhibition of the Notch pathway by podocyte-specific genetic ablation of the Notch coactivator RBP-Jκ or pharmacological blockade of γ-secretase reversed glomerular damage and re-established the filtration barrier [47]. Transgenic TGF-β1 overexpression cause podocyte injury, proteinuria and progressive glomerulosclerosis [48]. Moreover, Notch inhibition modulate TGF-β-mediated p53-dependent podocyte apoptosis [16]. In cultured human podocytes, TGF-β1, VEGF and high glucose activate Notch pahway and induce podocyte apoptosis [16], [49]. However, in these cells AngII did not induce apoptosis [50], and did not increase Jagged-1 production. In a rat model of diabetic nephropathy pharmacological inhibition of the Notch signaling ameliorated proteinuria [49], showing that podocyte-specific Notch inhibition could be a good therapeutic option for proteinuric diseases, characterized by podocyte loss by apoptosis.

Divergent Notch functionality has been described depending on cell type. In the vasculature Notch-3 regulates vascular tone and cell growth/apoptosis [51], [52]. In these cells AngII inhibited Notch-3 [51], while in tubular epithelial cells, neither TGF-β1 nor AngII modulate Notch-3. In the kidney, Notch-3 upregulation was only observed in renal progenitors in human glomeruloesclerosis [14], supporting the role of Notch in renal regeneration.

Understanding the fine regulation of the Notch system in kidney injury is necessary since Notch signaling may impact kidney regeneration in addition to injury. In adult kidneys a resident renal cell population with progenitor activity strongly expresses members of the Notch signaling pathway [53]. In folic acid-induced renal injury, Notch inhibition did not modify acute renal injury and creatinine levels (a marker of renal recovery), but ameliorated renal lesions and fibrosis, observed at 7 days [20]. Interestingly, Notch activation was detected only in proliferating cells. In a model of acute tubular necrosis induced by ischemia-reperfusion, treatment with the Notch ligand Delta-like-4 facilitated renal recovery by increasing cell proliferation [21]. These data suggest that the described beneficial effects of Notch inhibition could be due to the modulation of cell proliferation. Furthermore, Notch activation in human renal progenitors stimulates cell proliferation, whereas its downregulation is required for differentiation toward the podocyte lineage. Indeed, persistent Notch activation induced podocyte death by mitotic catastrophe [14]. In mouse models of focal segmental glomerulosclerosis, Notch inhibition reduced podocyte loss and ameliorated proteinuria during the initial phases of glomerular injury, but Notch inhibition in the regenerative phases of glomerular injury reduced progenitor cell proliferation and worsened proteinuria and glomerulosclerosis [14].

There is a lack of effective therapy for chronic renal diseases. The beneficial effect of Notch inhibition in experimental proteinuric glomerular diseases, including diabetic nephropathy, shows the importance of Notch activation in podocyte failure. However, we describe here that the Notch pathway is not involved in AngII-induced fibrotic events. AngII contributes to renal damage progression, by inducing fibrosis-related events, and its blockade retards renal disease progression in humans. Although there are some current clinical trials using the γ-secretase inhibitors for diseases as diverse as Alzheimer's and leukemia [47], our experimental studies does not support the potential beneficial effect of these drugs in AngII-mediate renal diseases. Our results show the complexity of the regulation of the Notch pathway in the kidney, and suggest that the involvement of this pathway in renal disease progression could be due to regulation of regeneration [14], [21] rather than by its contribution to fibrosis. Our findings clearly indicate that more studies are necessary to improve the actual therapeutic approaches to limit renal damage progression, before the use of the γ-secretase inhibitors for human diseases.

## Methods

### Ethics Statement

All experimental procedures were approved by the Animal Care and Use Committee of the IIS-Fundación Jimenez Diaz, according to the guidelines for ethical care of the European Community.

### Experimental models

The model of systemic infusion of AngII was done in 3-month-old male Normotensive Wistar-Kyoto (WKY, Criffa, Barcelona, Spain). AngII (Biochem) dissolved in saline was infused at the dose of 100 ng/kg/min by subcutaneous osmotic minipumps (Alza Corp) for different time periods (from 24 hours to 2 weeks; n = 8 animals per group). A control group of saline-infused rats of the same age was also studied (n = 8 animals). SHR male rats of 16 weeks of age were studied as control group normotensive WKY of the same age were used (n = 8 animals per group).

Diabetic nephropathy (DN) was induced by two streptozotocin (STZ) injections (50 mg/kg per day) or vehicle (0.01 mol/L citrate buffer pH 4.5) in 6 week-old normotensive Wistar-Kyoto rats which were studied after 6 weeks of diabetes (n = 10 animals per group). Insulin (1–4 IU subcutaneous, Insulatard NPH) was administrated weekly to prevent death from 7 days after administration of STZ, once all animals had blood glucose levels >400 mg/dl. Systolic blood pressure was measured monthly in conscious, restrained rats by the tail-cuff sphygmomanometer (NARCO, Biosystems). The average of three separate measurements was calculated at each time point. Albuminuria in 24 hour/urine samples was assessed by ELISA (Celltrend, Luckenwalde, Germany). The control group was the same as SHR rats.

The model of unilateral ureteral obstruction (UUO) was done in male C57BL/6 mice. The model was performed under isoflurane-induced anesthesia; the left ureter was ligated with silk (4/0) at two locations and cut between ligatures to prevent urinary tract infection (obstructed kidney), as described [38]. Some animals were treated with the AT1 antagonist Losartan (MSD, Spain; 10 mg/kg per day; drinking water), starting 1 day before UUO and continued for 5 days (n = 6 mice per group).

At the time of sacrifice, animals were anesthetized with 5 mg/kg xylazine (Rompun, Bayer AG) and 35 mg/kg ketamine (Ketolar, Fisher) and the kidneys perfused *in situ* with cold saline before removal. A piece of the kidney (2/3) was fixed, embedded in paraffin, and used for immunohistochemistry, and the rest was snap-frozen in liquid nitrogen for renal cortex RNA and protein studies. In UUO model, studies were done comparing both kidneys (contralateral and obstructed) in each mouse. In addition, a control group of sham-operated mice was also done, showing the same results than contralateral kidneys (data not shown).

### Cell cultured studies

Human renal proximal tubular epithelial cells (HK-2 cell line, ATCC CRL-2190) were grown in RPMI 1640 medium with 10% fetal bovine serum (FBS), 2 mmol/L glutamine, 100 U/mL penicillin, 100 μg/mL streptomycin, 5 μg/mL Insulin Transferrin Selenium (ITS) and 36 ng/mL hydrocortisone in 5% CO_2_ at 37°C. At 60–70% of confluence, cells were growth-arrested in serum-free medium for 24 hours before the experiments.

Human podocytes are an immortalized cell line, transfected with a temperature-sensitive SV40 gene construct and a gene encoding the catalytic domain of human telomerase [54]. At a permissive temperature of 33°C, the cells remain in an undifferentiated proliferative state, whereas raising the temperature to 37°C results in growth arrest and differentiation to the parental podocyte phenotype. Undifferentiated podocyte cultures were maintained at 33°C in RPMI 1640 medium with penicillin; streptomycin; insulin, transferrin, and selenite; and 10% FBS. Once cells had reached 70 to 80% confluence, they were cultured at 37°C for at least 14 days before use, when full differentiation had taken place. For experiments, cells were cultured in serum-free medium 24 hours before the addition of the stimuli and throughout the experiment.

Murine renal cortical fibroblasts (TFB cell line) originally obtained from Dr. Eric Neilson (Vanderbilt University) were grown in RPMI 1640 with 10% FBS, 2 mM glutamine, 100 U/ml penicillin and 100 μg/ml streptomycin in 5% CO_2_ at 37°C [55]. At 60–70% of confluence, cells were growth-arrested in serum-free medium for 24 hours before the experiments.

Cells were cultured in six-well plates, serum starved for 24 hours and treated with vehicle (PBS), recombinant human TGF-β1 (Peprotech), recombinant Ang II (Sigma) or recombinant human Jagged-1 (R&D systems) for 24 or 48 hours in serum-free medium. The γ-secretase inhibitor IX (DAPT, Calbiochem) was added together with TGF-β1 at 3×10^−8^ mol/L DAPT for 24 hours. DMSO, used as solvent, had no effect on cell viability and gene expression (not shown). Cells were used for protein or RNA studies, and the supernatants (cell-conditioned media) for TGF-β1 measurements.

### Protein studies

Cells were homogenized in lysis buffer (50 mmol/L Tris/HCl; 150 mol/L NaCl; 2 mmol/L EDTA; 2 mmol/L EGTA; 0.2% Triton X-100; 0.3% IGEPAL, 10 μl/mL protease inhibitors cocktail; 1 μl/mL PMSF, 1 μl/mL and 10 μl/mL orthovanadate) and then separated by SDS-polyacrylamide gel electrophoresis. Jagged-1 and EMT markers were determined in total protein extracts by western blot, 20 μg of proteins were loaded in each lane. Protein content was determined by the BCA method (Pierce, Rockford). The efficacy of protein transfer to the membranes was assessed by Red Ponceau staining (data not shown). To evaluate equal loading, membranes were stained with anti-GAPDH antibody. The autoradiographs were scanned using the GS-800 Calibrated Densitometer (Quantity One, Bio-Rad). The following primary antibodies were employed [dilution]: Jagged-1 (Santa Cruz, [1∶500]); Vimentin (R&D, [1/10000]); pan-Cytokeratin (Sigma-Aldrich, [1/10000]); GAPDH (Chemicon International, [1/5000]).

Paraffin-embedded kidney biopsy specimens were used for evaluation of Jagged-1 and Notch intracellular domain (NICD) staining. Specific biotinylated secondary antibodies were used, followed by streptavidin–horseradish peroxidase conjugate, and developed with diaminobenzidine. The following primary antibodies were employed [dilution]: Jagged-1 (Santa Cruz, [1∶100]); NICD (Abcam, [1∶300]). Briefly, 5 μm thick renal sections were deparaffinized and endogenous peroxidase was blocked by 3% H_2_O_2_ for 20 min. Then, the sections were incubated overnight at 4°C with specific primary antibodies. The specificity was checked by omission of primary antibodies.

For immunocytochemistry experiments, cells were grown on coverslips. After incubation, cells were fixed in paraformaldehyde 4% and permeabilized with 0.2% Triton-X100 for 10 min. After blocking with 3% BSA, they were incubated with primary antibodies: anti NICD (abcam, 1∶300) overnight at 4°C, followed by a AlexaFluor® 488 secondary antibody (Invitrogen) for 1 h. Nuclei were stained with 4′,6-Diamidino-2-phenyindole (DAPI). Absence of primary antibody was used as negative control. Samples were mounted in Mowiol 40–88 (Sigma-Aldrich) and examined by a Leica DM-IRB confocal microscope.

For the evaluation of TGF-β1 protein levels an ELISA kit from eBioscience was used, and TGF-β1 levels were quantified by comparison with a standard curve. In the *in vitro* studies, the conditioned media were collected to evaluate active TGF-β1 (as described above), and data were expressed as fold-change over untreated cells. In the different experimental models, renal TGF-β1 protein levels were evaluated in 0.1 μg/mL of total renal protein extracts, and data were expressed as fold-change the mean of value of the corresponding control animal in each model.

### Gene expression studies

Total RNA was isolated from cells with Trizol (Invitrogen). cDNA was synthesized from 2 μg of total RNA primed with random hexamer primers using the High capacity cDNA Archive Kit (Applied). Multiplex real time PCR was performed using Applied Biosystems expression assays (Taqman Fam fluorophore) as follows: Jagged1: Hs01070032_m1; Notch1: Hs 00413187_m1; Delta1: Hs01128541_m1, Notch3: Hs00194509_m1, Vimentin: Hs00185584_m1; MMP-9: Hs00234579_m1 PAI-1: Hs00167155_m1 and CTGF: Hs00170014_m1. Data were normalized to 18S eukaryotic ribosomal RNA: 4210893E (Vic). The mRNA copy numbers were calculated for each sample by the instrument software using Ct value (“arithmetic fit point analysis for the lightcycler”). Results were expressed in copy numbers, calculated relative to unstimulated cells after normalization against 18S.

### Statistical analysis

Results are expressed as n-fold increase over control as mean ± SEM. Differences between groups were assessed by Mann-Whitney test. p<0.05 was considered significant. Statistical analysis was conducted using the SPSS statistical software (version 11.0, Chicago, IL).

## Supporting Information

Figure S1 **AT1 antagonist treatment ameliorates renal damage in the model of unilateral ureteral obstruction in mice.** Animals were treated daily with the AT1 antagonist losartan, starting 1 day before unilateral obstruction, and animals were studied 5 days after obstruction. **A.** In obstructed kidneys there is a marked inflammatory cell infiltration that was diminished in Losartan-treated mice. The figure A shows of CD3 lymphocytes immunostaining of a representative animal of each group (magnification 200x). **B. Losartan downregulated proinflammatory factors.** The MCP-1 gene expression was evaluated by real time PCR. **C. Losarta-n diminished renal TGF-β1 protein levels.** TGF-β1 was determined by ELISA. Data is shown as mean ± SEM of 6 animals per group. *p<0.05 vs contralateral kidneys. # p<0.05 vs untreated.
(TIF)
