# Supplementary figures and images for: Angiotensin II Contributes to Renal Fibrosis Independently of Notch Pathway Activation
Source: PLoS One. 2012 Jul 9;7(7):e40490. doi: 10.1371/journal.pone.0040490 (PMC3392235; doi:10.1371/journal.pone.0040490)

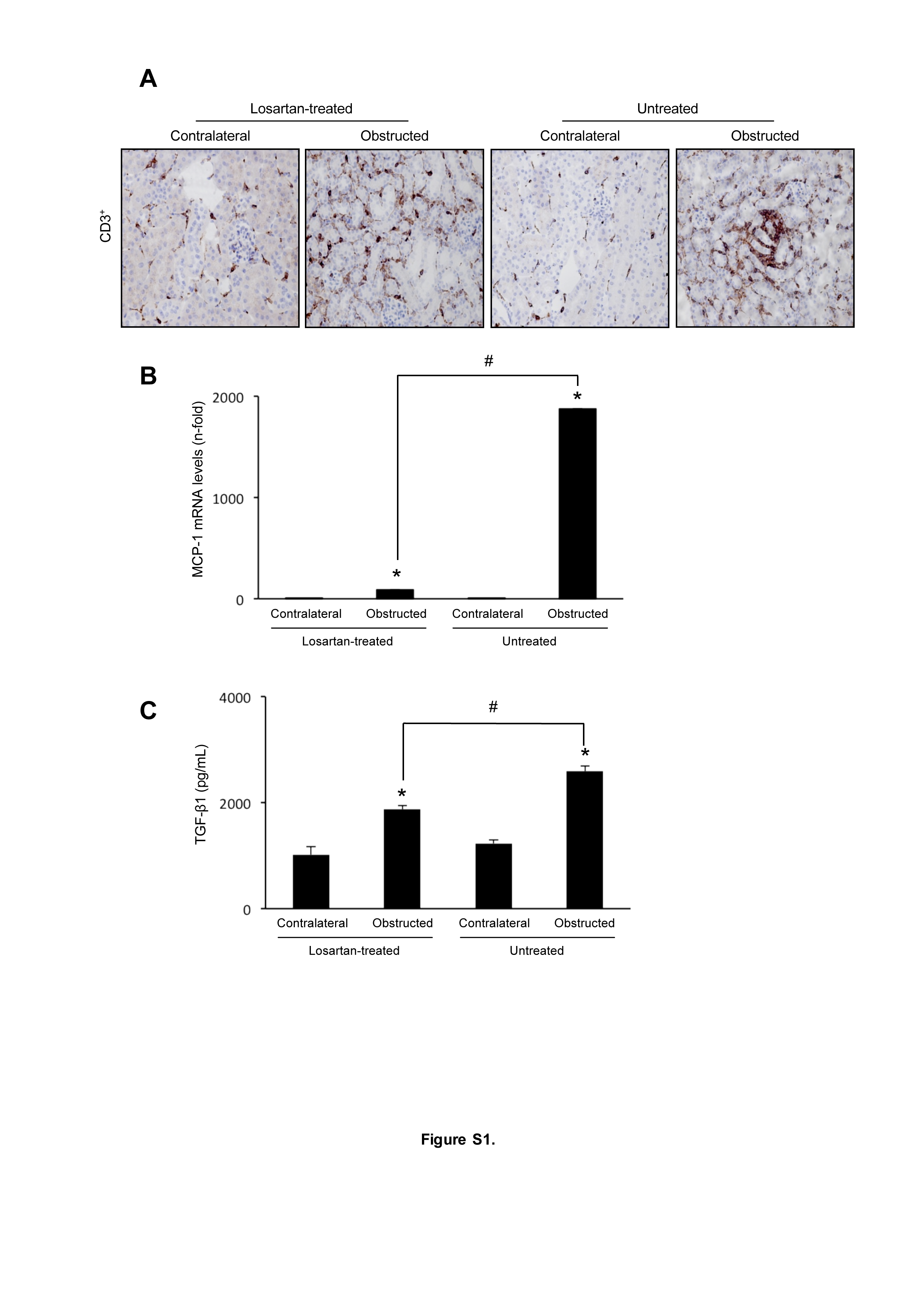

Supplement: Figure S1 — AT1 antagonist treatment ameliorates renal damage in the model of unilateral ureteral obstruction in mice. Animals were treated daily with the AT1 antagonist losartan, starting 1 day before unilateral obstruction, and animals were studied 5 days after obstruction. A. In obstructed kidneys there is a marked inflammatory cell infiltration that was diminished in Losartan-treated mice. The figure A shows of CD3 lymphocytes immunostaining of a representative animal of each group (magnification 200x). B. Losartan downregulated proinflammatory factors. The MCP-1 gene expression was evaluated by real time PCR. C. Losarta-n diminished renal TGF-β1 protein levels. TGF-β1 was determined by ELISA. Data is shown as mean ± SEM of 6 animals per group. *p<0.05 vs contralateral kidneys. # p<0.05 vs untreated. (TIF) [file pone.0040490.s001.tif]
